# Sweet Taste Receptor TAS1R2 Polymorphism (Val191Val) Is Associated with a Higher Carbohydrate Intake and Hypertriglyceridemia among the Population of West Mexico

**DOI:** 10.3390/nu8020101

**Published:** 2016-02-19

**Authors:** Omar Ramos-Lopez, Arturo Panduro, Erika Martinez-Lopez, Sonia Roman

**Affiliations:** 1 Department of Molecular Biology in Medicine, Civil Hospital of Guadalajara, “Fray Antonio Alcalde”, Guadalajara, Jalisco 44280, Mexico; os_mar6@hotmail.com (O.R.-L.); apanduro@prodigy.net.mx (A.P.); erikamtz27@yahoo.com.mx (E.M.-L.); 2 Health Sciences Center, University of Guadalajara, Guadalajara, Jalisco 44340, Mexico

**Keywords:** *TAS1R2* gene, Ile191Val polymorphism, carbohydrate intake, hypertriglyceridemia, West Mexico

## Abstract

Some high-carbohydrate diets may lead to obesity and multiple metabolic disorders, including hypertriglyceridemia (HTG). This lipid abnormality is considered an important risk factor for cardiovascular disease and type 2 diabetes. The sweet taste receptor *TAS1R2* polymorphism (Ile191Val) has been reported to be associated with carbohydrate intake. The aim of this study was to analyze the association of the *TAS1R2* gene polymorphism with carbohydrate intake and HTG among the population of West Mexico. In a cross-sectional study, 441 unrelated subjects were analyzed for *TAS1R2* genotypes (Ile/Ile, Ile/Val and Val/Val) by an allelic discrimination assay. Biochemical tests and a three-day food record were assessed. The Val/Val genotype carriers had a higher intake of total carbohydrates, fiber and servings of cereals and vegetables than the other genotype carriers. The Val/Val genotype conferred a higher risk for HTG than the Ile/Val and Ile/Ile genotypes (OR = 3.26, 95%CI 1.35–7.86, *p* = 0.006 and OR = 2.61, 95%CI 1.12–6.07, *p* = 0.02, respectively). Furthermore, the Val/Val genotype was associated with approximately 30% higher triglycerides compared with Ile/Val and Ile/Ile genotypes (β = 44.09, 95%CI 9.94–78.25, *p* = 0.01 and β = 45.7, 95%CI 10.85–80.54, *p* = 0.01, respectively). In conclusion, the Val/Val genotype of *TAS1R2* was associated with a higher carbohydrate intake and HTG.

## 1. Introduction

The traditional diet of the ancient Mexicans (AD 900-1521) provided essential nutrients, such as dietary fiber, polyunsaturated fatty acids, and antioxidants to the native population [1,2]. This traditional diet appears to have exerted genetic adaptations for regional food sources leading to an efficient use of nutrients [2]. However, in the last 30 years, the Mexican population has experienced a nutrition transition characterized by an excessive intake of processed foods [3]. Regarding this point, the current diet among the people of West Mexico is marked by a frequent consumption of industrially sweetened beverages containing high-fructose corn syrup, refried foods in oil or lard, red meat, and confectionary foods [4]. These food trends have changed the nutritional composition of the traditional diet by increasing the proportional amount of simple carbohydrates, saturated fatty acids and cholesterol [4,5]. It has been documented that the long-term consumption of this type of unbalanced diet is a crucial risk factor for the development of obesity, nonalcoholic steatohepatitis, liver cirrhosis and other nutrition-related diseases [6]. Currently, Mexico ranks as the most obese country in the world, with more than 70% of adults being classified as overweight or obese [7]. It has been reported that high carbohydrate diets (>55% of total energy) lead to obesity and multiple metabolic disorders including hypertriglyceridemia (HTG) [8,9,10,11,12]. High levels of serum triglycerides (TG) are a risk factor for the development of type 2 diabetes and liver cirrhosis [13,14]. In Mexico, HTG is also highly prevalent (>40%) [15], and both type 2 diabetes and cirrhosis are the leading causes of morbidity and mortality in the country [16].

Taste perception plays a key role in determining the individual food preferences and dietary habits [17]. In particular, sweet taste is a powerful factor influencing food acceptance [18]. At the molecular level, all sweet taste perception is mediated by the sweet taste receptor, a heterodimer of the G-protein–coupled receptors TAS1R2-TAS1R3 [19]. Nevertheless, TAS1R2 is considered the specific component to sweet taste perception because TAS1R3, which also responds to l-amino acids, is involved in the detection of umami taste when it dimerizes with the TAS1R1 receptor [20]. In addition to gene expression in the tongue and palate, TAS1R2 is also expressed in other body tissues that regulate metabolism and energy homeostasis. Thus, genetic variations in the TAS1R2 receptor may contribute to inter-individual differences in dietary intake [17,21].

The *TAS1R2* gene is located on chromosome 1 [22]. Several single-nucleotide polymorphisms (SNPs) in this gene have been identified; one is located in exon 3 that causes a nucleotide substitution at position 571 (Adenine/Guanine, A571G, rs35874116) [23]. This non-synonymous polymorphism leads to the amino acid substitution at position 191 (Isoleucine/Valine, Ile191Val), which has been associated with the regular consumption of sugars in healthy and diabetic subjects [24]. However, the influence of the *TAS1R2* gene in carbohydrate intake among the Mexican population is currently unknown. Therefore, the aim of this study was to analyze the association of the Ile191Val *TAS1R2* gene polymorphism with carbohydrate intake and HTG among the population of West Mexico.

## 2. Experimental Section

### 2.1. Study Population

In a cross-sectional/analytical study, a total of 441 unrelated Mestizos subjects of both genders and over 18 years of age were included. The study was conducted at the Department of Molecular Biology in Medicine, Civil Hospital of Guadalajara “Fray Antonio Alcalde” in Guadalajara, Jalisco, Mexico. Exclusion criteria were women who were pregnant or breastfeeding, smokers and individuals with chronic sinus problems, subjects taking any prescribed medication that might affect taste perception and lipid levels, and those who reported consuming a special diet that restricted carbohydrates or calories in the last six months.

### 2.2. Anthropometric Measurements

Height measurement was determined by using a clinical scale with a stadiometer (Rochester Clinical Research, New York, NY, USA) during the patient’s visit. Body Mass Index (BMI, kg/m^2^) was determined by electrical bioimpedance using an INBODY 3.0 instrument (Analyzer Body Composition, Biospace, Korea).

### 2.3. Dietary Assessment

A 3-day food record was used to assess daily intake of macronutrients. Each subject was instructed on how to complete this tool, including two weekdays and one weekend day. The food records were coded by a trained registered dietitian using the Nutrikcal computer program (Nutrikcal VO^®^, México), which is based on the Mexican System of Food and Equivalents [5]. Macronutrient intakes were averaged over the 3-day food records.

### 2.4. Biochemical Tests

Ten-milliliter blood samples were drawn by venipuncture after a 12-h fast and separated into two aliquots; one for DNA isolation and another for determination of biochemical test. Blood tests included glucose, total cholesterol (TC), TG, and high-density lipoprotein cholesterol (HDL-c). Low-density lipoprotein cholesterol (LDL-c) was calculated using the Friedewald formula [25], and very low-density lipoprotein cholesterol (VLDL-c) concentration was calculated as TC − (LDL-c + HDL-c). Dry chemistry assay was used to determine all biochemical tests on a Vitros 250 Analyzer (Ortho Clinical Diagnostics, Johnson & Johnson Co, Rochester, NY, USA). For quality control purposes, we used a pooled human serum and a commercial control serum (Ortho Clinical Diagnostics, Johnson & Johnson Co, Rochester, NY, USA) to account for imprecision and the inaccuracy of the biochemical measurements. The intra-assay variability (coefficient of variation, CV%) was estimated by ten repeated determinations of the control serum in the same analytical session. Inter-assay CV% for each variable was calculated by the mean values of control serum measured in five analytical sessions. When necessary, the serum was diluted with bovine serum albumin according to the manufacturers' instructions. The criteria for considering HTG were TG > 150 mg/dL, TC < 200 mg/dL and LDL-c < 130 mg/dL [26].

### 2.5. TAS1R2 Genotyping

DNA was extracted from leukocytes by a modified salting-out method [27]. The Ile191Val *TAS1R2* gene polymorphism (rs35874116) was determined by a TaqMan allelic discrimination assay (Assay Number C_55646_20, Applied Biosystems, Foster City, CA, USA) in a 96-well format and read on a Step One Plus thermocycler (Applied Biosystems, Foster City, CA, USA). DNA was used at a final concentration of 70 ng. PCR conditions were 95 °C for 10 min and 40 cycles of denaturation at 92 °C for 15 s and annealing/extension at 60 °C, for 1 min. *TAS1R2* genotyping was verified using positive controls of the DNA samples corresponding to the three possible genotypes in each 96-well plate as well as re-running 10% of the total samples, which were 100% concordant.

### 2.6. Statistical Analyses

Quantitative values are expressed as mean ± standard deviation (SD), whereas qualitative variables were expressed as number and percentage. A formula for a cross-sectional/analytical study [28] was used to calculate the sample size, which resulted in 290 subjects based on the allelic frequencies in the general population reported by Eny *et al.* [24]. Based on the sample size, the statistical power of the study was 80% (β = 0.20) with a statistical reliability of 95% (α = 0.05). Statistical differences for quantitative variables were analyzed by one-way ANOVA test and adjusted for age, gender and BMI. Subsequently, *post hoc* tests were run to define intergroup differences according to the homogeneity of variances. Bonferroni’s test assuming equal variances and Dunnett’s T3 test assuming unequal variances were used. Qualitative variables and Hardy-Weinberg equilibrium (HWE) were analyzed by chi-square test. Odds ratio (OR), logistic (adjusted OR) and linear regression were performed to test the association of the *TAS1R2* gene polymorphism with HTG. A *p*-value < 0.05 was considered significant. Statistical analyzes were performed by using Epi-info TM7 (CDC, Atlanta, GA, USA) and IBM SPSS software version 20 for Windows (IBM Inc., Armonk, NY, USA).

### 2.7. EthicalGuidelines

The study protocol complied with the ethical guideline for the 2013 Declaration of Helsinki and was approved by the local Hospital Ethical Committee. All participants filled out a written informed consent.

## 3. Results

### 3.1. Distribution of the TAS1R2 Gene Polymorphism and Characteristics of the Study Population

As shown in Table 1, the genotype frequencies of the *TAS1R2* gene polymorphism were Ile/Ile (56.9%), Ile/Val (37.4%) and Val/Val (5.7%), whereas the frequencies of the Ile allele and Val allele were 75.6% and 24.4%, respectively. The distribution of genotypes was concordant with the Hardy-Weinberg Equilibrium (*p* = 0.90). In regards to the demographic characteristics of the participants, no significant differences between the variables of age, gender, BMI and the *TAS1R2* genotype category were found.

### 3.2. Daily Dietary Intake of the Study Population

Table 2 and Table 3 depict the daily dietary intake of the study group according to the *TAS1R2* genotype. Among the three *TAS1R2* genotype groups, the Val/Val carriers comparatively had a higher daily intake of total carbohydrates (*p* = 0.01) and fiber (*p* = 0.002) than the other genotype carriers (Table 2). Additionally, they had a higher daily intake of vegetables (*p* = 0.005) and cereal (*p* = 0.01) servings than those who were non-Val/Val carriers (Table 3).

### 3.3. Biochemical Profile

The effect of the *TAS1R2* genotype on the biochemical profile of the study group is depicted in Table 4. The Val/Val genotype carriers had a significantly higher serum levels of TG (*p* = 0.02) than the other genotype carriers.

### 3.4. Lipid Profile and Association of TAS1R2 Genotype with Hypertriglyceridemia

As shown in Table 5, the percentage of subjects with HTG was greater among the Val/Val carriers compared to the non-HTG group (10.9% *vs.* 4.1%). Furthermore, the Val/Val genotype conferred a higher risk for HTG in comparison with the Val/Ile, Ile/Ile and both genotypes combined.

These results were confirmed with logistic regression tests (OR = 3.81, 95%CI 1.47–9.86, *p* = 0.006; OR = 2.49, 95%CI 1.02–6.04, *p* = 0.04 and OR = 2.89, 95%CI 1.21–6.88, *p* = 0.01, respectively). Furthermore, by means of a linear regression test, an increase of approximately 30% higher serum TG was associated with the Val/Val genotype compared to the same genotype combinations mentioned before (β = 44.09, 95%CI 9.94–78.25, *p* = 0.01; β = 45.7, 95%CI 10.85–80.54, *p* = 0.01 and β = 48.34, 95%CI 8.42–88.26, *p* = 0.01, respectively).

## 4. Discussion

The onset, progression and clinical outcome of several chronic diseases driven by carbohydrate and lipoprotein abnormalities are known to be influenced by genetic polymorphisms interacting with environmental factors [29]. In the context of this study, the genetic architecture of the Mexican population is characterized by an admixture of three paternal lineages consisting of Amerindian, European (Caucasian) and African ancestry, denoted as Mestizos, with an unequal inter-regional distribution [30,31,32,33]. Therefore, among the Mexican population, it is expected that the distribution of the risk and protector alleles of certain lipid transporters, taste receptors and metabolizing enzymes may vary due to the degree of ancestral inheritance and prevalence of regional environmental factors [34,35,36,37,38,39]. In this study, the Val/Val risk genotype was prevalent in 5.7% among the studied population of West Mexico. This relatively low frequency was similar to the pattern of Val/Val genotype distribution (9.8%) reported in white population from Canada [24] and was consistent with the predominant Caucasian ancestral component estimated in Mexican-Mestizos from the State of Jalisco [30,31]. Conversely, higher frequencies of the Val/Val genotype have been reported in African descendant population [32]. However, further investigation is required to establish the pattern of distribution of the *TAS1R2* gene and its impact on sweet taste perception and carbohydrate intake in other regions of Mexico, particularly among the Native Amerindians who are exposed to absolutely different gene-environmental interactions [34]

Studies on the TAS1R2 receptor have focused on the effects of its genetic variations on sweet taste perception [40], sugar or carbohydrate intake *per se* [24,39,40] and the prevalence of dental caries in children and adults [41,42,43], whereas the association with dyslipidemia have been less explored in humans. In this study, we highlight the association of the Val/Val genotype with a higher carbohydrate intake and HTG among a Mestizo population of West Mexico. The significantly higher carbohydrate intake observed among the Val/Val genotype carriers may be attributed to a corresponding increase in the average daily intake of cereals. One plausible explanation may be that genetically speaking, the Ile191Val polymorphism resides in the predicted first large extracellular domain of the TAS1R2 receptor, which hypothetically contains the ligand-binding site for carbohydrates and dipeptide sweeteners [44,45,46]. Particularly, this functional domain also displays significant genetic polymorphism and haplotype diversity presumably associated with the evolutionary adaptation humans have made by natural sugar nutrients [23]. This complex diversity is notable when revising studies that report divergent results. For example, an association between the rs12033832 (G>A) SNP with higher sucrose taste thresholds and sugar intake in overweight subjects was recently documented [39]; whereas, for the rs35874116 SNP, a lower carbohydrate intake among Val allele carriers in comparison to the Ile homozygotes was reported in Canadian subjects [24]. Moreover, these discrepancies may also be caused by ethnic differences between populations, gene-environment interactions, study design, mode of inheritance and food culture [47].

Regarding food culture, the traditional Mexican diet contained a wide variety of wild and domestic crops, as well as game and domesticated animals dated as far back as Paleolithic and Neolithic pre-Hispanic times [1,2]. One staple cereal highly consumed with a great variety of Mexican dishes is the “tortilla”, a low-fat, maize-derived product that provides a significant amount of dietary fiber and calcium [1,4]. Nonetheless, the current-day obesogenic environment in Mexico has promoted the substitution of the natural, traditional “tortilla” for high-fat industrialized cereals in the form of sweet bread and pastries [4,5]. The progressive shift in the last 500 years from a traditional native diet towards a westernized lifestyle may be just one of the many factors related to the obesity epidemic and derived comorbidities based on the existence of the recent genetic and cultural admixture of the Mexicans [1]. Thus, given the occurrence of a high copy number of the amylase 1 (*AMY1*) gene [48] and the differential distribution of the apolipoprotein E2 (*APOE2*) and *APOE4* polymorphisms [34], foods that are high in simple sugars, saturated fat and cholesterol may be detrimental to both Amerindians and Mexican-Mestizos with a stronger Amerindian lineage [1]. Likewise, this evolutionary discordance may also occur with the Ile191Val *TAS1R2* polymorphisms in modern-day Mexicans that acquire new dietary habits.

As for lipid metabolism, in this study, we found an association between the Val/Val genotype and high serum TG, as well as an increased risk for HTG compared to the other genotypes. This dyslipidemia could be due to the higher carbohydrate intake observed among the Val/Val carriers since they were the only group with a carbohydrate consumption of >55% of the total energy [49]. It has been proposed that many of the changes induced by high-carbohydrate diets in lipid metabolism could be interpreted as a shift from fatty acid oxidation to triglyceride synthesis, which is mediated by an increase in insulin concentrations [50]. The principal mechanisms involved in carbohydrate-induced HTG include *de novo* lipogenesis, activation of stearoyl-CoA desaturase activity, accelerated VLDL-triglyceride secretion, reduced muscle fatty acid oxidation and low clearance of lipids from the blood [8,9,10,11,12,13,51,52,53,54,55,56,57,58]. Furthermore, high-carbohydrate diets have also been associated with an increased risk of type 2 diabetes, coronary heart disease, nonalcoholic fatty liver disease, and some types of cancer [59,60,61,62].

Alongside, a higher fat intake was observed in the Val homozygotes, although it was not statistically significant because all study groups consistently consumed fatty foods. These dietary habits are in accordance with previous nutritional studies [4,5] and with those that report a genetic susceptibility associated with fatty food preference [36] and HTG [63,64] among the population of West Mexico. Additionally, one of the adverse effects of HTG is hypo-alpha-lipoproteinemia or lower than normal HDL-c levels [65]. Interesting, in this study, we found no differences in the HDL-c levels among the *TAS1R2* genotypes that were low despite a trend in overgrowth of levels in Val/Val carriers. This observation may be explained by the fact that several loci diminish HDL-c levels among the Mexican population, such as the ATP-binding cassette transporter member A1 (*ABCA1*), hepatic lipase gene (*LIPC*), cholesteryl ester transfer protein (*CETP*) and LOC55908 [66,67]. These genes may also interact with environmental factors including physical activity [68,69] that require investigation in our population. Moreover, further studies are needed to establish the association of other common genetic SNPs of leptin and its receptor, the glucose transporter type 2 and the dopamine receptor D_2_ with sweet preferences and frequent sugar consumption that has been consistently reported in distinct populations [70,71,72].

In regards to the nutritional guidelines for the treatment of dyslipidemias, one of the most fundamental approaches is to increase dietary fiber intake [73,74]. However, in this study, the Val homozygotes had a high intake of dietary fiber due to the consumption of cereals and vegetable servings, yet they also had a higher frequency of HTG. Similar results were found in another study where an elevation of serum TG was observed after a high-carbohydrate intake even when the diet was composed of high-fiber whole foods [75]. In this study, the average fiber intake in all study groups (including the Val/Val carriers) was lower than the recommended 14 g/1000 cal to reduce lipid levels [76]. The beneficial effect of dietary fiber occurs when it is combined with a low-fat diet [8]. However, in this study, the diet of all study groups was high in fat as mentioned before. Additionally, it has been documented that BMI >28 kg/m^2^ is a risk factor known to increase the sensitivity to lipid and lipoprotein alterations in response to high-carbohydrate diets [8]. This fact is in agreement with our study because the Val/Val genotype carriers were the only group with a BMI over 28 kg/m^2^. Thus, these findings support that HTG is a multi-causal metabolic disorder where several factors are involved. Moreover, genetic variations in lipid proteins have been associated with HTG in our population, such as *APOE2* and fatty acid-binding protein 2 (*FABP2*) that may also be playing an important role in this condition [35,63,64].

## 5. Conclusions

In conclusion, to the best of our knowledge, this study is the first to report the prevalence and association of Val191Val polymorphism with high carbohydrate intake and dyslipidemia in a population of West Mexico, data that may be representative of the Caucasian component predominant in several regions of the country. However, in the context of the obesity epidemic and high prevalence of HTG in Mexico, the detection of the *TAS1R2* Val/Val genotype along with other gene polymorphisms may be an auxiliary tool for the identification of high-risk groups and prediction of resistance or responsiveness to dietary treatments. The prevention of HTG is a matter of considerable impact because left unattended, HTG has been known to induce insulin resistance and eventually liver cirrhosis or type 2 diabetes in susceptible individuals [29].

To date, despite the negative impact of the high prevalence of type 2 diabetes and liver cirrhosis on public health, no interventions have been effective in reducing the rates of obesity and derived comorbidities in our population [1,77]. With the current knowledge that many genes influence food intake and eating behaviors, the success of these strategies may largely depend on the individual’s genetic characteristics [36]. Therefore, new research in the field of nutrigenetics and genomics is required to develop genome-based intervention strategies that include designing region-tailored diets with nutrients that are compatible with the ancestral gene-environmental interactions of each population [1].

## Figures and Tables

**Table 1:** Comparison of the subject characteristics by *TAS1R2* genotype (*n* = 441).

Variable	*TAS1R2* Genotype			*p*-Value
	Ile/Ile	Ile/Val	Val/Val	
Number of subjects, *n* (%)	251 (56.9)	165 (37.4)	25 (5.7)	-
Age (years)	41.8 ± 14.1	41.4 ± 14.2	41.1 ± 14.7	0.94
Gender (F/M)	(146/105)	(87/78)	(14/11)	0.55
BMI (kg/m^2^)	27.4 ± 5.4	27.8 ± 5.3	28.7 ± 5.9	0.20

Average values are expressed as mean ± SD. Gender is represented as frequency. F: female, M: male, BMI: Body mass index.

**Table 2:** Average daily intake of macronutrients by *TAS1R2* genotype.

Macronutrient	*TAS1R2* Genotype			*p*-Value
	Ile/Ile *N* = 251	Ile/Val *N* = 165	Val/Val *N* = 25	
Calories	2069 ± 587	2017 ± 627	2287 ± 627	0.12
Protein (%)	16.1 ± 3.5	16.9 ± 4.3	16.5 ± 4.3	0.53
Protein (g)	83.4 ± 26.5	83.9 ± 29.6	89.2 ± 23.7	0.61
Total fat (%)	29.1 ± 7.1	31.6 ± 9.4	32.4 ± 9.3	0.20
Total fat (g)	73.8 ± 28.4	72.2 ± 32.2	74.2 ± 30.2	0.81
Total carbohydrates (%)	52.4 ± 10.5	53.1 ± 11.3	58.5 ± 8.8	0.04 *
Total carbohydrates (g)	273 ± 102.4	265.2 ± 98.1	332.7 ± 102.6	0.01 **
Fiber (g)	17.4 ± 11.3	19.1 ± 12.9	26.3 ± 12.1	0.002 **

Average values are expressed as mean ± SD. * Val/Val genotype *vs.* Ile/Ile genotype ** Val/Val genotype *vs.* Ile/Val and Ile/Ile genotypes.

**Table 3:** Average daily intakes of food group servings by *TAS1R2* genotype.

Food Group	*TAS1R2* Genotype			*p*-Value
	Ile/Ile *N* = 251	Ile/Val *N* = 165	Val/Val *N* = 25	
Sugars	5.4 ± 5.1	5.3 ± 4.7	5.5 ± 4.1	0.96
Meat	6.4 ± 3.3	6.7 ± 4.0	5.9 ± 3.0	0.52
Fruits	1.5 ± 1.9	1.7 ± 2.1	2.0 ± 1.9	0.30
Vegetables	2.5 ± 2.3	2.5 ± 2.2	4.2 ± 4.7	0.005 **
Fats	4.4 ± 3.4	4.2 ± 3.7	5.5 ± 3.6	0.19
Milk	1.0 ± 1.1	0.9 ± 1.1	0.9 ± 0.8	0.96
Legumes	0.6 ± 0.9	0.7 ± 0.9	0.9 ± 1.1	0.21
Cereals	9.5 ± 4.9	8.8 ± 4.7	11.8 ± 5.3	0.01 ***

Average values are Number of Servings expressed as mean ± SD. ** Val/Val genotype *vs.* Ile/Val and Ile/Ile genotypes; *** Val/Val genotype *vs.* Ile/Val genotype.

**Table 4:** Comparison of biochemical profile by *TAS1R2* genotype.

Variable	*TAS1R2* Genotype			*p*-Value
	Ile/Ile *N* = 251	Ile/Val *N* = 165	Val/Val *N* = 25	
Glucose (mg/dL)	93.8 ± 14.6	93.6 ± 13.2	97.1 ± 25.2	0.56
TC (mg/dL)	180.8 ± 46.8	184.9 ± 46.2	188.1 ± 86.1	0.61
TG (mg/dL)	149 ± 82	150 ± 75	194 ± 100	0.02 **
HDL-c (mg/dL)	40.4 ± 11.5	41.4 ± 12.6	43.2 ± 14.9	0.46
LDL-c (mg/dL)	110.1 ± 39.6	118.6 ± 38.6	105.1 ± 56.4	0.13
VLDL-c (mg/dL)	32.5 ± 23.8	32.6 ± 22.6	42.1 ± 22.1	0.26

Average values are expressed as mean ± SD. TC: Total Cholesterol; TG: Triglycerides; HDL-c: High-Density Lipoprotein cholesterol; LDL-c: Low-Density Lipoprotein cholesterol; VLDL-c: Very Low Lipoprotein cholesterol. ** Val/Val genotype *vs.* Ile/Val and Ile/Ile genotypes.

**Table 5:** Association of the *TAS1R2* genotype with hypertriglyceridemia.

*TAS1R2* Genotypes	Non-HTG *n* (%)	HTG *n* (%)	Genotype Comparison	Odds Ratio (95%CI)	*p*-Value
Ile/Ile	193 (56.8)	58 (57.4)	Val/Val *vs.* Ile/Val	3.26 (1.35–7.86)	0.006
Ile/Val	133 (39.1)	32 (31.7)	Val/Val *vs.* Ile/Ile	2.61 (1.12–6.07)	0.02
Val/Val	14 (4.1)	11 (10.9)	Val/Val *vs.* Ile/Val and Ile/Ile	2.84 (1.24–6.48)	0.009

Frequencies of HTG are expressed as percentage of each *TAS1R2* genotype. HTG: Hypertriglyceridemia. The criteria for considering HTG were TG > 150 mg/dL, TC < 200 mg/dL and LDL-c < 130 mg/dL).

## References

[1] Roman S., Ojeda-Granados C., Ramos-Lopez O., Panduro A. (2015). Genome-based nutrition: An intervention strategy for the prevention and treatment of obesity and nonalcoholic steatohepatitis. World J. Gastroenterol..

[2] Román S., Ojeda-Granados C., Panduro A. (2013). Genética y evolución de la alimentación de la población en México. Rev. Endocrinol. Nutr..

[3] Barrera-Cruz A., Rodríguez-González A., Molina-Ayala M.A. (2013). Escenario actual de la obesidad en México. Rev. Med. Inst. Mex. Segurol. Soc..

[4] Ramos-López O., Román S., Ojeda-Granados C., Sepúlveda-Villegas M., Martínez-López E., Torres-Valadez R., Trujillo-Trujillo E., Panduro A. (2013). Patrón de ingesta alimentaria y actividad física en pacientes hepatópatas en el Occidente de México. Rev. Endocrinol. Nutr..

[5] Ramos-López O., Ojeda-Granados C., Román S., Panduro A. (2013). Influencia genética en las preferencias alimentarias. Rev. Endocrinol. Nutr..

[6] Flores M., Macias N., Rivera M., Lozada A., Barquera S., Rivera-Dommarco J., Tucker K.L. (2010). Dietary patterns in Mexican adults are associated with risk of being overweight or obese. J. Nutr..

[7] Rtveladze K., Marsh T., Barquera S., Sanchez R.L.M., Levy D., Melendez G., Webber L., Kilpi F., McPherson K., Brown M. (2014). Obesity prevalence in Mexico: Impact on health and economic burden. Public Health Nutr..

[8] Parks E.J., Hellerstein M.K. (2000). Carbohydrate-induced hypertriacylglycerolemia: Historical perspective and review of biological mechanisms. Am. J. Clin. Nutr..

[9] Rutledge J.C., Hyson D.A., Garduno D., Cort D.A., Paumer L., Kappagoda C.T. (1999). Lifestyle modification program in management of patients with coronary artery disease: The clinical experience in a tertiary care hospital. J. Cardiopulm. Rehabil..

[10] Hudgins L.C. (2000). Effect of high-carbohydrate feeding on triglyceride and saturated fatty acid synthesis. Proc. Soc. Exp. Biol. Med..

[11] Hudgins L.C., Hellerstein M., Seidman C., Neese R., Diakun J., Hirsch J. (1996). Human fatty acid synthesis is stimulated by a eucaloric low fat, high carbohydrate diet. J. Clin. Investig..

[12] Fried S.K., Rao S.P. (2003). Sugars, hypertriglyceridemia, and cardiovascular disease. Am. J. Clin. Nutr..

[13] Subramanian S., Chait A. (2012). Hypertriglyceridemia secondary to obesity and diabetes. Biochim. Biophys. Acta.

[14] Schult A., Eriksson H., Wallerstedt S., Kaczynski J. (2011). Overweight and hypertriglyceridemia are risk factors for liver cirrhosis in middle-aged Swedish men. Scand. J. Gastroenterol..

[15] Aguilar-Salinas C.A., Canizales-Quinteros S., Rojas-Martínez R., García-García E., Olaiz-Fernández G., Gómez-Pérez F.J., Tusié-Luna M.T. (2007). Colaboraciones exitosas entre tres instituciones mexicanas en el estudio de las dislipidemias, la obesidad y la diabetes. Gac. Méd. Méx..

[16] Gómez-Dantés O., Sesma S., Becerril V.M., Knaul F.M., Arreola H., Frenk J. (2011). Sistema de salud de México. Salud Publica Mex..

[17] Garcia-Bailo B., Toguri C., Eny M., El-Sohemy A. (2009). Genetic variation in taste and its influence on food selection. OMICS.

[18] Bachmanov A.A., Bosak N.P., Floriano W.B., Inoue M., Li X., Lin C., Murovets V.O., Reed D.R., Zolotarev V.A., Beauchamp G.K. (2011). Genetics of sweet taste preferences. Flavour Fragr. J..

[19] Cui M., Jiang P., Maillet E., Max M., Margolskee R.F., Osman R. (2006). The heterodimeric sweet taste receptor has multiple potential ligand binding sites. Curr. Pharm. Des..

[20] Toda Y., Nakagita T., Hayakawa T., Okada S., Narukawa M., Imai H., Ishimaru Y., Misaka T. (2013). Two distinct determinants of ligand specificity in T1R1/T1R3 (the umami taste receptor). J. Biol. Chem..

[21] Drewnowski A. (1997). Taste preferences and food intake. Annu. Rev. Nutr..

[22] Liao J., Schultz P.G. (2003). Three sweet receptor genes are clustered in human chromosome 1. Mamm. Genome.

[23] Kim U.K., Wooding S., Riaz N., Jorde L.B., Drayna D. (2006). Variation in the human TAS1R taste receptor genes. Chem. Senses.

[24] Eny K.M., Wolever T.M., Corey P.N., El-Sohemy A. (2010). Genetic variation in TAS1R2 (Ile191Val) is associated with consumption of sugars in overweight and obese individuals in 2 distinct populations. Am. J. Clin. Nutr..

[25] Tremblay A.J., Morrissette H., Gagné J.M., Bergeron J., Gagné C., Couture P. (2004). Validation of the Friedewald formula for the determination of low-density lipoprotein cholesterol compared with beta-quantification in a large population. Clin. Biochem..

[26] Secretaría de Salud Norma Oficial Mexicana NOM-037-SSA2–2002, Para la Prevención, Tratamiento y Control de las Dislipidemias. Http://www.dof.gob.mx/nota_detalle.php?codigo=5285372&fecha=22/01/2013NOM.

[27] Miller S.A., Dykes D.D., Polesky H.F. (1988). A simple salting out procedure for extracting DNA from human nucleated cells. Nucleic Acids Res..

[28] Aguilar-Barojas S. (2005). Fórmulas para el cálculo de la muestra en investigaciones de salud. Salud en Tabasco.

[29] Ramos-Lopez O., Martinez-Lopez E., Roman S., Fierro N.A., Panduro A. (2015). Genetic, metabolic and environmental factors involved in the development of liver cirrhosis in Mexico. World J. Gastroenterol..

[30] Rangel-Villalobos H., Muñoz-Valle J.F., González-Martín A., Gorostiza A., Magaña M.T., Páez-Riberos L.A. (2008). Genetic admixture, relatedness, and structure patterns among Mexican populations revealed by the Y-chromosome. Am. J. Phys. Anthropol..

[31] Martínez-Cortés G., Salazar-Flores J., Haro-Guerrero J., Rubi-Castellanos R., Velarde-Félix J.S., Muñoz-Valle J.F., López-Casamichana M., Carrillo-Tapia E., Canseco-Avila L.M., Bravi C.M. (2013). Maternal admixture and population structure in Mexican-Mestizos based on mtDNA haplogroups. Am. J. Phys. Anthropol..

[32] International HapMap Consortium (2003). The International HapMap Project. Nature.

[33] Rubi-Castellanos R., Martínez-Cortés G., Muñoz-Valle J.F., González-Martín A., Cerda-Flores R.M., Anaya-Palafox M., Rangel-Villalobos H. (2009). Pre-Hispanic Mesoamerican demography approximates the present-day ancestry of Mestizos throughout the territory of Mexico. Am. J. Phys. Anthropol..

[34] Aceves D., Ruiz B., Nuño P., Roman S., Zepeda E., Panduro A. (2006). Heterogeneity of apolipoprotein E polymorphism in different Mexican populations. Hum. Biol..

[35] Martinez-Lopez E., Garcia-Garcia M.R., Gonzalez-Avalos J.M., Maldonado-Gonzalez M., Ruiz-Madrigal B., Vizmanos B., Hernandez-Nazara Z., Roman S., Panduro A. (2013). Effect of Ala54Thr polymorphism of FABP2 on anthropometric and biochemical variables in response to a moderate-fat diet. Nutrition.

[36] Ramos-Lopez O., Panduro A., Martinez-Lopez E., Fierro N.A., Ojeda-Granados C., Sepulveda-Villegas M., Roman S. (2015). Genetic variant in the CD36 Gene (rs1761667) is associated with higher fat intake and high serum cholesterol among the population of West Mexico. J. Nutr. Food Sci..

[37] Ramos-Lopez O., Roman S., Martinez-Lopez E., Gonzalez-Aldaco K., Ojeda-Granados C., Sepulveda-Villegas M., Panduro A. (2015). Association of a novel TAS2R38 haplotype with alcohol intake among Mexican-Mestizo population. Ann. Hepatol..

[38] Roman S., Zepeda-Carrillo E.A., Moreno-Luna L.E., Panduro A. (2013). Alcoholism and liver disease in Mexico: Genetic and environmental factors. World J. Gastroenterol..

[39] Salguero M.L., Leon R.E., Santos A., Roman S., Segura-Ortega J.E. (2005). The role of FABP2 gene polymorphism in alcoholic cirrhosis. Hepatol. Res..

[40] Dias A.G., Eny K.M., Cockburn M., Chiu W., Nielsen D.E., Duizer L., El-Sohemy A. (2015). Variation in the TAS1R2 Gene, Sweet Taste Perception and Intake of Sugars. J. Nutrigenet. Nutrigenomics.

[41] Izakovicova Holla L., Borilova Linhartova P., Lucanova S., Kastovsky J., Musilova K., Bartosova M., Kukletova M., Kukla L., Dusek L. (2015). GLUT2 and TAS1R2 Polymorphisms and Susceptibility to Dental Caries. Caries Res..

[42] Haznedaroğlu E., Koldemir-Gündüz M., Bakır-Coşkun N., Bozkuş H.M., Çağatay P., Süsleyici-Duman B., Menteş A. (2015). Association of sweet taste receptor gene polymorphisms with dental caries experience in school children. Caries Res..

[43] Robino A., Bevilacqua L., Pirastu N., Situlin R., Di Lenarda R., Gasparini P., Navarra C.O. (2015). Polymorphisms in sweet taste genes (TAS1R2 and GLUT2), sweet liking, and dental caries prevalence in an adult Italian population. Genes Nutr..

[44] Nie Y., Vigues S., Hobbs J.R., Conn G.L., Munger S.D. (2005). Distinct contributions of T1R2 and T1R3 taste receptor subunits to the detection of sweet stimuli. Curr. Biol..

[45] Pin J.P., Galvez T., Prézeau L. (2003). Evolution, structure, and activation mechanism of family 3/C G-protein-coupled receptors. Pharmacol. Ther..

[46] Xu H., Staszewski L., Tang H., Adler E., Zoller M., Li X. (2004). Different functional roles of T1R subunits in the heteromeric taste receptors. Proc. Natl. Acad. Sci. USA.

[47] Salanti G., Southam L., Altshuler D., Ardlie K., Barroso I., Boehnke M., Cornelis M.C., Frayling T.M., Grallert H., Grarup N. (2009). Underlying genetic models of inheritance in established type 2 diabetes associations. Am. J. Epidemiol..

[48] Mejía-Benítez M.A., Bonnefond A., Yengo L., Huyvaert M., Dechaume A., Peralta-Romero J., Klünder-Klünder M., García Mena J., El-Sayed Moustafa J.S., Falchi M. (2015). Beneficial effect of a high number of copies of salivary amylase AMY1 gene on obesity risk in Mexican children. Diabetologia.

[49] Parks E.J. (2001). Effect of dietary carbohydrate on triglyceride metabolism in humans. J. Nutr..

[50] Roberts R., Bickerton A.S., Fielding B.A., Blaak E.E., Wagenmakers A.J., Chong M.F., Gilbert M., Karpe F., Frayn K.N. (2008). Reduced oxidation of dietary fat after a short term high-carbohydrate diet. Am. J. Clin. Nutr..

[51] Chong M.F., Fielding B.A., Frayn K.N. (2007). Metabolic interaction of dietary sugars and plasma lipids with a focus on mechanisms and de novo lipogenesis. Proc. Nutr. Soc..

[52] Hudgins L.C., Hellerstein M.K., Seidman C.E., Neese R.A., Tremaroli J.D., Hirsch J. (2000). Relationship between carbohydrate-induced hypertriglyceridemia and fatty acid synthesis in lean and obese subjects. J. Lipid Res..

[53] Schwarz J.M., Linfoot P., Dare D., Aghajanian K. (2003). Hepatic de novo lipogenesis in normoinsulinemic subjects consuming high-fat, low-carbohydrate and low-fat, high-carbohydrate isoenergetic diets. Am. J. Clin. Nutr..

[54] Chong M.F., Hodson L., Bickerton A.S., Roberts R., Neville M., Karpe F., Frayn K.N., Fielding B.A. (2008). Parallel activation of de novo lipogenesis and stearoyl-CoA desaturase activity after 3 d of high-carbohydrate feeding. Am. J. Clin. Nutr..

[55] Mittendorfer B., Sidossis L.S. (2001). Mechanism for the increase in plasma triacylglycerol concentrations after consumption of short-term, high-carbohydrate diets. Am. J. Clin. Nutr..

[56] Marques-Lopes I., Ansorena D., Astiasaran I., Forga L., Martínez J.A. (2001). Postprandial de novo lipogenesis and metabolic changes induced by a high-carbohydrate, low-fat meal in lean and overweight men. Am. J. Clin. Nutr..

[57] Parks E.J., Parks E.J. (2002). Changes in fat synthesis influenced by dietary macronutrient content. Proc. Nutr. Soc..

[58] Baum C.L., Brown M. (2000). Low-fat, high-carbohydrate diets and atherogenic risk. Nutr. Rev..

[59] Schernhammer E.S., Hu F.B., Giovannucci E., Michaud D.S., Colditz G.A., Stampfer M.J., Fuchs C.S. (2005). Sugar-sweetened soft drink and risk of pancreatic cancer in two prospective cohorts. Cancer Epidemiol. Biomark. Prev..

[60] Larsson S.C., Bergkvist L., Wolk A. (2006). Consumption of sugar and sugar-sweetened foods and the risk of pancreatic cancer in a prospective study. Am. J. Clin. Nutr..

[61] Schulze M.B., Manson J.E., Ludwig D.S., Colditz G.A., Stampfer M.J., Willett W.C., Hu F.B. (2004). Sugar-sweetened beverages, weight gain, and incidence of type 2 diabetes in young and middle-aged women. JAMA.

[62] Malik V.S., Schulze M.B., Hu F.B. (2006). Intake of sugar-sweetened beverages and weight gain: A systematic review. Am. J. Clin. Nutr..

[63] Martinez-Lopez E., Curiel-Lopez F., Hernandez-Nazara A., Moreno-Luna L.E., Ramos-Marquez M.E., Roman S., Panduro A. (2015). Influence of ApoE and FABP2 polymorphisms and environmental factors in the susceptibility to gallstone disease. Ann. Hepatol..

[64] Hernández-Nazará Z.H., Ruiz-Madrigal B., Martínez-López E., Roman S., Panduro A. (2008). Association of the epsilon 2 allele of APOE gene to hypertriglyceridemia and to early-onset alcoholic cirrhosis. Alcohol Clin. Exp. Res..

[65] Bo S., Cavallo-Perin P., Gentile L., Repetti E., Pagano G. (2001). Low HDL-cholesterol: A component of the metabolic syndrome only in the presence of fasting hypertriglyceridemia in type 2 diabetic patients. Diabetes Metab..

[66] Weissglas-Volkov D., Aguilar-Salinas C.A., Nikkola E., Deere K.A., Cruz-Bautista I., Arellano-Campos O., Muñoz-Hernandez L.L., Gomez-Munguia L., Ordoñez-Sánchez M.L., Reddy P.M. (2013). Genomic study in Mexicans identifies a new locus for triglycerides and refines European lipid loci. J. Med. Genet..

[67] Villarreal-Molina M.T., Aguilar-Salinas C.A., Rodríguez-Cruz M., Riaño D., Villalobos-Comparan M., Coral-Vazquez R., Menjivar M., Yescas-Gomez P., Königsoerg-Fainstein M., Romero-Hidalgo S. (2007). The ATP-binding cassette transporter A1 R230C variant affects HDL cholesterol levels and BMI in the Mexican population: Association with obesity and obesity-related comorbidities. Diabetes.

[68] Ossoli A., Gomaraschi M., Franceschini G., Calabresi L. (2014). Genetic determinants of HDL metabolism. Curr. Med. Chem..

[69] Ahmad T., Chasman D.I., Buring J.E., Lee I.M., Ridker P.M., Everett B.M. (2011). Physical activity modifies the effect of LPL, LIPC, and CETP polymorphisms on HDL-C levels and the risk of myocardial infarction in women of European ancestry. Circ. Cardiovasc. Genet..

[70] Mizuta E., Kokubo Y., Yamanaka I., Miyamoto Y., Okayama A., Yoshimasa Y., Tomoike H., Morisaki H., Morisaki T. (2008). Leptin gene ad leptin receptor gene polymorphisms are associated with sweet preference and obesity. Hypertens. Res..

[71] Eny K.M., Wolever T.M., Fontaine-Bisson B., El-Sohemy A. (2008). Genetic variant in the glucose transporter type 2 is associated with higher intakes of sugars in two distinct populations. Physiol. Genomics.

[72] Eny K.M., Corey P.N., El-Sohemy A. (2009). Dopamine D2 receptor genotype (C957T) and habitual consumption of sugars in a free-living population of men and women. J. Nutrigenet. Nutrigenomics.

[73] Anderson J.W. (2000). Dietary fiber prevents carbohydrate-induced hypertriglyceridemia. Curr. Atheroscler. Rep..

[74] Jenkis D.J., Kendall C.W., Vuksan V., Vidgen E., Parker T., Faulkner D., Mehling C.C., Garsetti M., Testolin G., Cunnane S.C. (2002). Soluble fiber intake at a dose approved by the US Food and Drug Administration for a claim of health benefits: Serum lipid risk factors for cardiovascular disease assessed in a randomized controlled crossover trial. Am. J. Clin. Nutr..

[75] Parks E.J., Krauss R.M., Christiansen M.P., Neese R.A., Hellerstein M.K. (1999). Effects of a low-fat, high-carbohydrate diet on VLDL-triglyceride assembly, production, and clearance. J. Clin. Investig..

[76] Anderson J.W., Baird P., Davis R.H., Ferreri S., Knudtson M., Koraym A., Waters V., Williams C.L. (2009). Health benefits of dietary fiber. Nutr. Rev..

[77] Panduro A., Zacarias Castillo R. (2014). ¿Estamos incidiendo en el manejo médico-nutricional del paciente obeso en Mexico?. Rev. Mex. Endocrinol. Metabol. Nutr..

